# Detection of Oral Bacterial DNA in Abdominal Aortic Aneurysm and Its Microbial Associations

**DOI:** 10.3390/ijms27104396

**Published:** 2026-05-14

**Authors:** Kota Shimizu, Fukashi Serizawa, Daijirou Akamatsu, Sakae Saito, Yuichi Aoki, Michihisa Umetsu, Shunya Suzuki, Norinobu Ogasawara, Takashi Kamei

**Affiliations:** 1 Division of Vascular Surgery, Department of Surgery, Tohoku University Graduate School of Medicine, Sendai 980-8575, Japan; daijiro.akamatsu.c4@tohoku.ac.jp (D.A.); michihisa.umetsu.e1@tohoku.ac.jp (M.U.); shunya.suzuki.d3@tohoku.ac.jp (S.S.); norinobu.ogasawara.a3@tohoku.ac.jp (N.O.); takashi.kamei.a8@tohoku.ac.jp (T.K.); 2 Division of Vascular Surgery, Japanese Red Cross Ishinomaki Hospital, Ishinomaki 986-0861, Japan; fukashi.serizawa.a6@tohoku.ac.jp; 3 Division of Genomics, Tohoku Medical Megabank Organization, Tohoku University, Sendai 980-0872, Japan; sakae.saito.b6@tohoku.ac.jp (S.S.); yuichi.aoki.e3@tohoku.ac.jp (Y.A.)

**Keywords:** abdominal aortic aneurysm, 16S rRNA gene sequencing, oral microbiota, periodontal bacteria, aneurysmal wall, intraluminal thrombus

## Abstract

Abdominal aortic aneurysm (AAA) is a life-threatening disease for which no definitive medical therapy has been established, partly because its underlying mechanisms remain incompletely understood. Given accumulating evidence suggesting microbial involvement in vascular inflammation, we conducted a detection-based investigation to identify bacterial DNA in aneurysmal tissues. We performed 16S ribosomal RNA (rRNA) gene sequencing of the aneurysmal wall, intraluminal thrombus, feces, saliva, and dental plaque collected from 32 patients undergoing open surgical repair of non-infectious AAA. Based on the sequencing data, diversity analyses were performed for each sample to characterize bacterial composition, and exploratory statistical analyses were conducted to examine associations between patient characteristics and the relative abundance of bacterial taxa. Oral-associated genera were frequently detected in aneurysm-derived samples, including *Prevotella* in 78%, *Leptotrichia* in 81%, and *Capnocytophaga* in 38% of aneurysmal wall or thrombus samples, whereas their detection in fecal samples was limited. Beta diversity analysis demonstrated significant compositional differences between fecal and oral samples (permutational multivariate analysis of variance [PERMANOVA], *p* < 0.01). These findings demonstrate the presence of bacterial DNA in aneurysmal tissues and provide descriptive evidence of microbial signatures in AAA.

## 1. Introduction

Abdominal aortic aneurysm (AAA) is a potentially fatal vascular disease characterized by localized dilation of the abdominal aorta. Despite its high risk of rupture and mortality, no pharmacological therapy has been established to date. One of the main barriers to drug development is the incomplete understanding of its pathogenesis. While AAA shares some features with atherosclerosis—such as inflammatory cell infiltration and foam cell formation—it exhibits distinct pathological changes, including degradation of the extracellular matrix (ECM), loss of vascular smooth muscle cells (VSMCs), and elastic fiber destruction, primarily in the medial and adventitial layers [1]. Among known risk factors, smoking is most strongly associated with AAA development and progression, contributing to chronic inflammation and matrix degradation through matrix metalloproteinase (MMP) activation and immune cell recruitment [2].

In recent years, increasing evidence has suggested that bacterial infection may contribute to AAA pathogenesis. Bacterial DNA, including that of *Helicobacter cinaedi*, has been detected in aneurysmal wall tissues of patients with non-infectious AAA [3]. These findings were based on high-sensitivity detection methods such as real-time quantitative polymerase chain reaction (qPCR) and 16S ribosomal RNA (rRNA) gene sequencing, indicating the presence of bacterial genetic material even in the absence of overt infection [3,4,5]. Experimental studies have also shown that antibiotic treatment can suppress aneurysm formation and progression, supporting a potential role for bacteria in modulating disease development [6,7].

Among the potential sources of such bacterial signals, the gut has been frequently considered. In murine models, alterations in the gut microbiota influence AAA formation and expansion [8,9]. In humans, 16S rRNA gene sequencing and PCR-based analyses have detected gut-derived bacterial DNA in aneurysmal walls and peripheral blood [4,5], suggesting that bacterial components can reach the aneurysmal tissue.

More recently, attention has shifted to the potential involvement of oral microbiota. Epidemiological studies have demonstrated associations between periodontitis and AAA prevalence [10,11]. In addition, bacterial DNA from oral pathogens such as *Fusobacterium nucleatum* and *Prevotella* species has been detected in aneurysmal tissues [12,13]. These pathogens are known to form biofilms—structured bacterial communities embedded in extracellular polymeric substances—that enable long-term persistence within tissues and resistance to host immune clearance [14,15,16].

Although accumulating evidence implicates both gut and oral microbiota in AAA pathogenesis, few studies have simultaneously assessed these microbial communities in relation to the aneurysmal wall and intraluminal thrombus. We hypothesized that a comprehensive microbiome analysis across multiple sample types would provide insight into the potential routes of bacterial translocation and the mechanisms by which bacteria may contribute to AAA progression. In this study, we performed 16S rRNA gene sequencing of aneurysmal wall, intraluminal thrombus, feces, saliva, and dental plaque samples obtained from patients undergoing open AAA repair. The primary objective of this study was to detect bacterial DNA in aneurysmal tissues and to characterize microbial composition across multiple sample types. Secondary analyses were exploratory and aimed to examine associations between clinical characteristics and bacterial abundance.

## 2. Results

### 2.1. Patient Demographics

A total of 32 patients were included. The median age was 70 years (interquartile range [IQR]: 65–75), and all patients were male. The median aneurysm diameter at surgery was 50 mm (IQR: 50–57 mm), with 88% (28 cases) presenting with fusiform aneurysms. A smoking history was present in 97% (31 patients), with 7 of them being current smokers. The median Brinkman Index score was 755. Other atherosclerotic risk factors included hypertension in 25 patients (78%), dyslipidemia in 22 patients (69%), and diabetes mellitus in 7 patients (22%). In addition, periodontitis was observed in 24 patients (75%) (Table 1). No patients included in this study showed clinical findings suggestive of infectious (mycotic) aneurysm, including fever or elevated inflammatory markers.

### 2.2. Sample Numbers

Aneurysmal wall samples were obtained from all 32 patients. Intraluminal thrombus samples were obtained from 31 patients; one patient had an “empty aneurysm” with no visible thrombus formation. Fecal samples were available for 28 patients, dental plaque for 20, and saliva for 24. Missing samples were due to preoperative bowel inactivity, skipped dental exams, or edentulism (Table 2).

### 2.3. Sequencing Summary and Amplicon Sequence Variant (ASV) Generation

To investigate the bacterial composition present in each sample obtained from patients, we performed 16S rRNA gene analysis. Comprehensive sequencing of the V4 region of the bacterial 16S rRNA gene yielded a total of 7.79 million paired-end reads. The median number of read pairs per sample was 28,343 (IQR: 25,207–31,430) for aneurysmal wall, 30,577 (IQR: 23,435–49,892) for intraluminal thrombus, 64,225 (IQR: 58,871–73,651) for feces, 82,920 (IQR: 69,479–92,614) for dental plaque, and 64,664 (IQR: 56,690–77,234) for saliva. The maximum read pair counts were 56,222, 56,279, 89,330, 150,242, and 98,844, respectively.

Subsequently, we applied quality-based filtering using DADA2, which included trimming of sequences, merging of paired-end reads, construction of ASVs via error-model-based denoising, and chimera removal. Representative sequences were then obtained for taxonomic classification. The median number of sequences after chimera removal was 1115 (IQR: 852–1991) for aneurysmal wall, 1818 (IQR: 1096–3583) for intraluminal thrombus, 54,506 (IQR: 49,571–62,539) for feces, 70,742 (IQR: 37,977–80,916) for dental plaque, and 55,223 (IQR: 45,647–64,040) for saliva. The maximum number of sequences after chimera removal was 13,890 for aneurysmal wall, 7797 for intraluminal thrombus, 75,238 for feces, 133,380 for dental plaque, and 85,466 for saliva.

**Figure 1:**
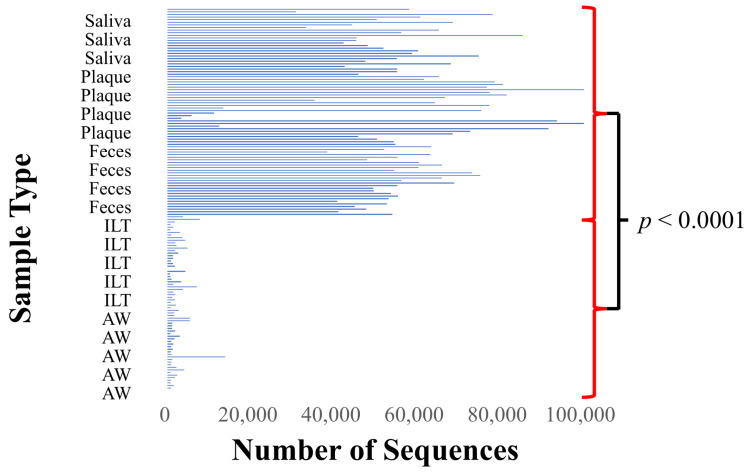
Number of high-quality sequencing reads after chimera removal. Bar chart showing the number of high-quality reads retained after chimera filtering for each sample across five sample types: saliva, dental plaque, feces, intraluminal thrombus, and aneurysmal wall. Samples from aortic tissues (aneurysmal wall and intraluminal thrombus) exhibited significantly lower read counts compared to oral and fecal samples. Abbreviations: ILT = intraluminal thrombus; AW = aneurysmal wall.

The number of sequences after chimera removal obtained from aneurysmal wall and thrombus samples was significantly lower than that from fecal and oral samples (Figure 1). Taxonomic analysis was subsequently performed on sequences that remained after noise removal.

### 2.4. α-Diversity

**Figure 2:**
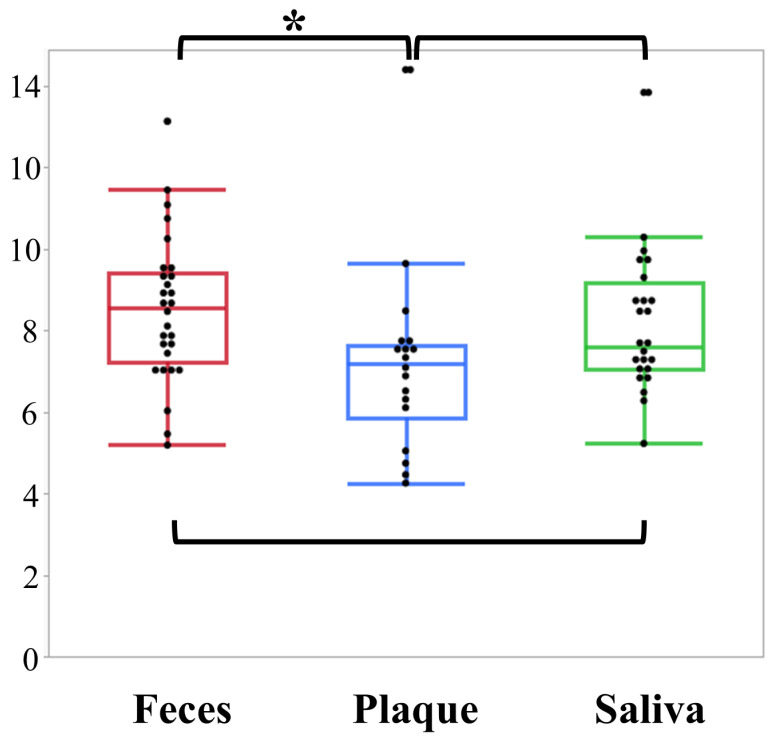
α-diversity analysis based on Faith’s PD in each sample type. Faith’s PD is shown for each sample group, with each dot representing an individual sample. Intergroup comparisons were performed using the Kruskal–Wallis test followed by the Steel–Dwass post hoc test for multiple comparisons. Microbial diversity in dental plaque was significantly lower than that in fecal samples (*p* = 0.02), whereas no significant differences were observed between dental plaque and saliva or between fecal and saliva samples (*p* = 0.12 and *p* = 0.60, respectively). *: *p* < 0.05.

Figure 2 presents the results of the α-diversity analysis. Among the specimens obtained from the aneurysmal wall and intraluminal thrombus, only one sample from each tissue type yielded more than 10,000 effective sequencing reads; therefore, these samples were excluded from the analysis. In addition, dental plaque and saliva samples with fewer than 10,000 valid reads were excluded. As a result, a total of 28 fecal, 18 dental plaque, and 24 saliva specimens were included in the final α-diversity analysis.

Faith’s phylogenetic diversity (Faith’s PD) differed significantly among the groups (Kruskal–Wallis test, *p* = 0.02). Post hoc multiple comparisons using the Steel–Dwass method demonstrated that microbial diversity in dental plaque was significantly lower than that in fecal samples (*p* = 0.02). In contrast, no significant differences were observed between dental plaque and saliva or between fecal and saliva microbiota (*p* = 0.12 and *p* = 0.60, respectively).

### 2.5. β-Diversity

**Figure 3:**
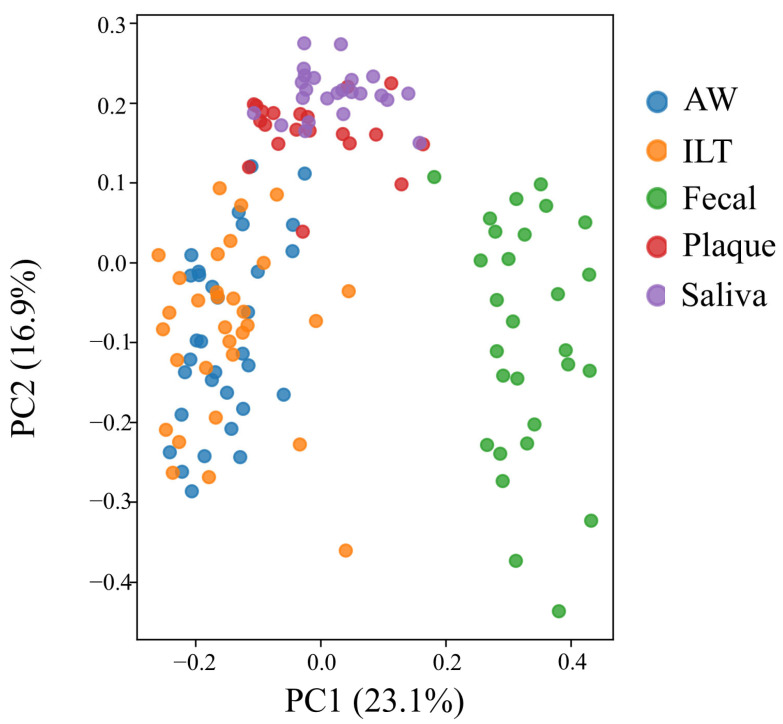
β-diversity analysis of microbial communities across sample types. Principal coordinate analysis (PCoA) based on Jaccard distance showing the distribution of microbial community composition in aneurysmal wall, intraluminal thrombus, feces, saliva, and dental plaque samples. Fecal samples were clearly separated from oral samples (saliva and dental plaque), whereas vascular samples showed dispersed distributions without forming distinct clusters. Statistical significance was assessed by permutational multivariate analysis of variance (PERMANOVA), which demonstrated significant differences in overall microbial community composition among sample types (pseudo-F = 22.24, *p* = 0.0001).

Figure 3 presents the results of the β-diversity analysis. Principal coordinate analysis (PCoA) based on Jaccard distance was performed to enable comparison across all sample types, including low-biomass vascular specimens (aneurysmal wall and intraluminal thrombus). Fecal samples were clearly separated from oral samples (saliva and dental plaque), whereas vascular samples (aneurysmal wall and intraluminal thrombus) showed dispersed distributions without forming distinct clusters. No clear separation was observed between dental plaque and salivary samples. Permutational multivariate analysis of variance (PERMANOVA) based on Jaccard distance revealed significant differences in overall microbial community composition among sample types (pseudo-F = 22.24, *p* = 0.0001).

### 2.6. Taxonomic Composition of Each Sample Type

Bacterial DNA was detected in aneurysmal samples from all patients across all taxonomic levels, from phylum to species, indicating that microbial signals were consistently present throughout the study cohort.

Bacterial presence/absence and relative abundance were analyzed at the genus and species levels. Because bacterial DNA was detected in all patients, associations between detection and demographic factors could not be assessed.

#### 2.6.1. Genus Level

**Figure 4:**
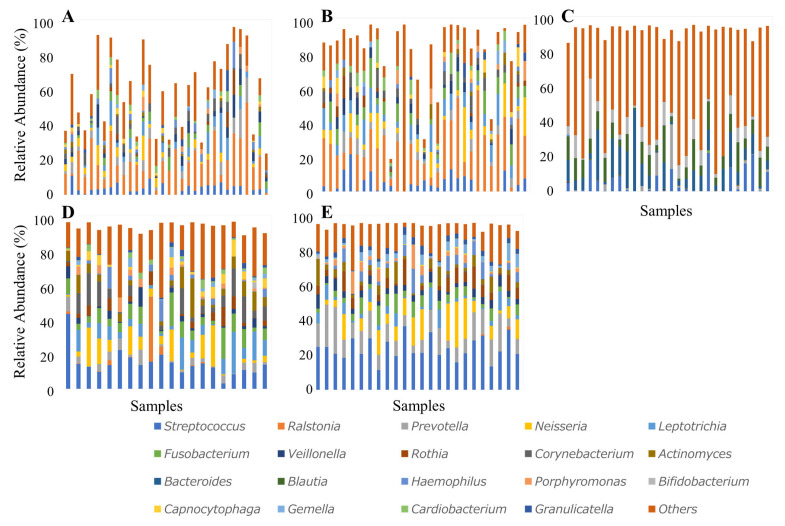
Genus-level relative abundance profiles across five sample types. Stacked bar plots showing the genus-level bacterial composition in (**A**) aneurysmal wall, (**B**) intraluminal thrombus, (**C**) feces, (**D**) dental plaque, and (**E**) saliva. Each bar represents one patient. The top genera are color-coded, with remaining genera grouped under “Others.” Taxonomic groups labeled as “No taxonomic information” were excluded from the plots.

In the aneurysmal wall, *Ralstonia* was detected in all samples. *Streptococcus* and *Leptotrichia*, typical oral bacteria, were detected in 78% of samples. In the intraluminal thrombus, *Ralstonia* was found in 98% and *Leptotrichia* in 81% of samples. Gut commensals such as *Streptococcus*, *Bacteroides*, *Blautia*, and *Bifidobacterium* were detected in all fecal samples. *Streptococcus*, *Prevotella*, *Leptotrichia*, *Fusobacterium*, and *Rothia* were detected in all oral samples (plaque and saliva, Figure 4A–E).

**Figure 5:**
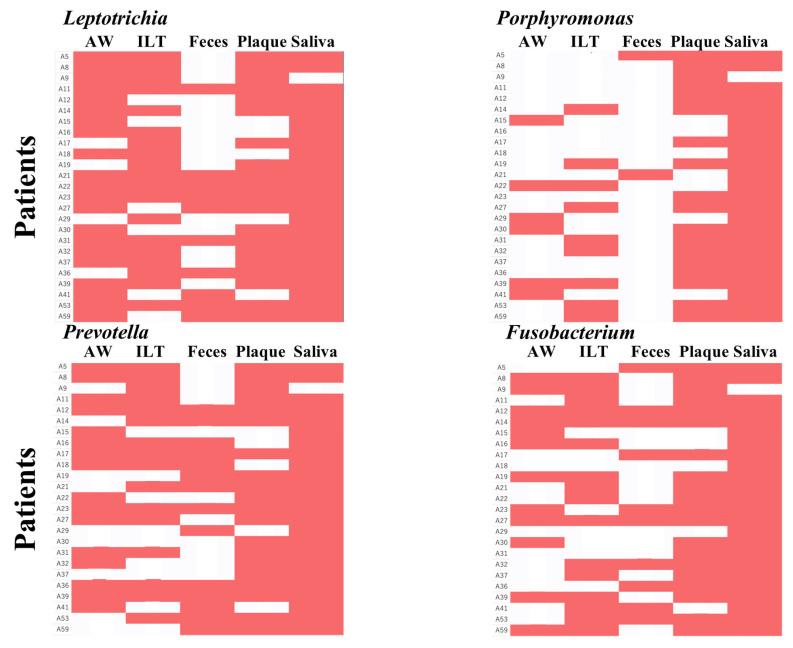
Heatmap of bacterial presence/absence at the genus level across patients and sample types. A heatmap was generated with patients on the y-axis and sample types (aneurysmal wall, intraluminal thrombus, feces, dental plaque, and saliva) on the x-axis. For each bacterial genus, detection is shown in red and absence in white. Bacterial genera detected in the aneurysmal wall or intraluminal thrombus were also detected in the corresponding oral samples (dental plaque or saliva).

Furthermore, when periodontal pathogen–associated genera such as *Leptotrichia*, *Porphyromonas*, *Prevotella*, and *Fusobacterium* were detected in aneurysm-derived samples (aneurysmal wall or intraluminal thrombus), they were also detected in the corresponding oral samples (dental plaque or saliva) from the same patients (Figure 5).

#### 2.6.2. Species Level

**Figure 6:**
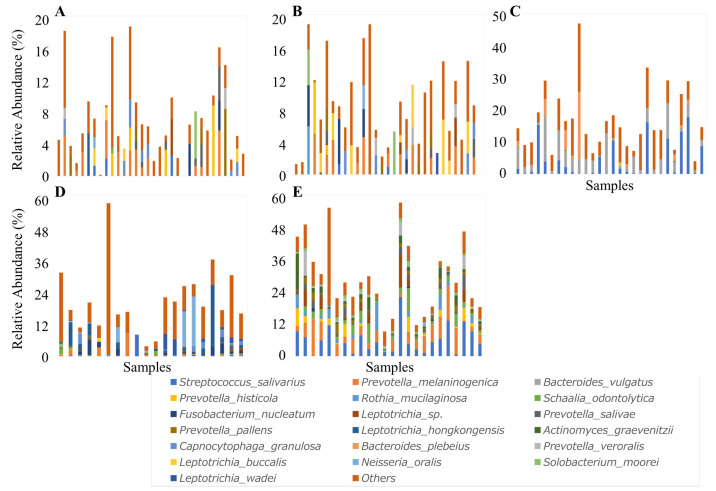
Species-level relative abundance profiles across five sample types. Stacked bar plots depicting the relative abundance of bacterial species in (**A**) aneurysmal wall, (**B**) intraluminal thrombus, (**C**) feces, (**D**) dental plaque, and (**E**) saliva. Frequently detected species are color-coded. Each bar corresponds to a patient. Species for which no taxonomic information was available were excluded from the plots.

In the aneurysmal wall, *Prevotella melaninogenica* and *Capnocytophaga granulosa* were found in 38% and 22% of samples, respectively. In the intraluminal thrombus, *Prevotella melaninogenica* was found in 35% of samples, and *Leptotrichia buccalis* in 26%. In feces, *Bacteroides vulgatus* and *Streptococcus salivarius* were detected in 100% and 97% of samples, respectively. In plaque samples, *Prevotella melaninogenica* (91%), *Capnocytophaga granulosa* (74%), and *Fusobacterium nucleatum* (65%) were detected. Saliva samples universally contained *Streptococcus salivarius*, and *Prevotella melaninogenica* (93%), *Prevotella salivae* (96%), and *Prevotella pallens* (93%) were detected in over 90% of samples (Figure 6).

**Figure 7:**
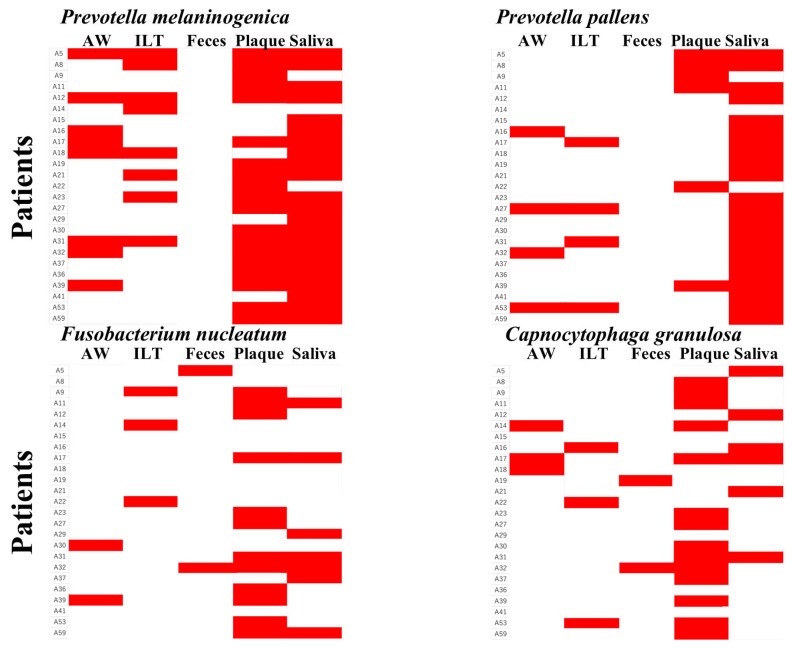
Heatmap of bacterial presence/absence at the species level across patients and sample types. Patients are shown on the y-axis, and sample types (aneurysmal wall, intraluminal thrombus, feces, dental plaque, and saliva) are displayed on the x-axis. For each bacterial species, detection is indicated in red and absence in white. *Prevotella melaninogenica* was detected in the corresponding dental plaque or saliva samples in all but one case, in which it was identified in the aneurysmal wall or intraluminal thrombus. *Prevotella pallens* was detected in the corresponding oral samples in all cases in which it was detected in the aneurysm-derived samples.

Among cases in which the periodontal bacterium *Prevotella melaninogenica* was detected in aneurysm-derived samples, all but one also showed detection in the corresponding oral samples from the same patients. *Prevotella pallens* was detected in the oral samples in all cases in which it was identified in aneurysm-derived samples. Although *Fusobacterium nucleatum* was not detected in the corresponding oral samples in one case from the aneurysmal wall and two cases from the intraluminal thrombus, it was also absent in the fecal samples from these patients. *Capnocytophaga granulosa* was not detected in the oral samples of one patient in whom it was present in the intraluminal thrombus, and it was similarly absent in the fecal sample from the same patient (Figure 7).

### 2.7. Exploratory Associations

**Figure 8:**
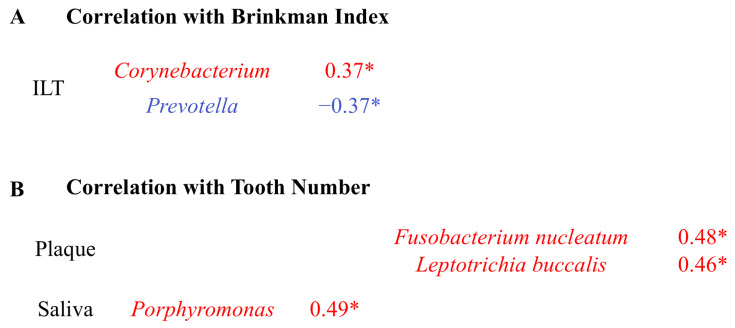
Correlation between patient characteristics and relative abundance of bacterial taxa. Correlations between the number of remaining teeth (**A**) and the Brinkman Index (**B**) and the relative abundance of bacterial taxa are shown. Only significant correlations (*p* < 0.05) are displayed. Numbers indicate Spearman’s correlation coefficients, and color indicates the direction of correlation (red, positive; blue, negative). *: *p* < 0.05.

**Figure 9:**
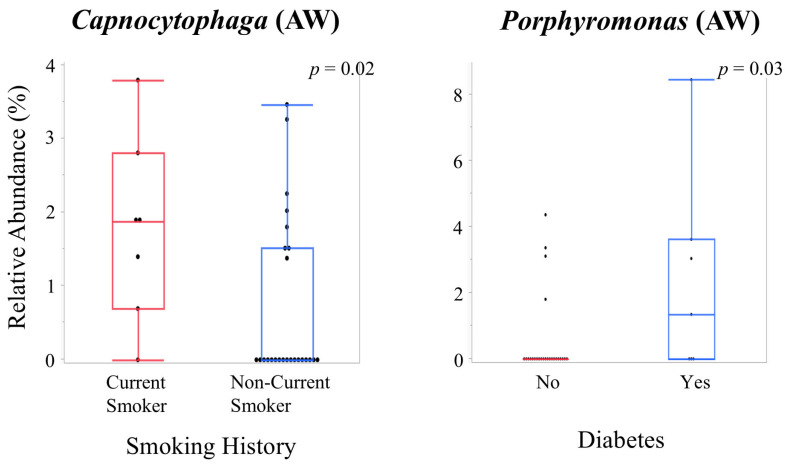
Comparison of relative abundance of bacterial genera according to patient characteristics. Boxplots showing the relative abundance of bacterial genera stratified by smoking history or diabetes status. Group comparisons were performed using the Wilcoxon rank-sum test. Statistical significance is indicated by *p*-values.

**Figure 10:**
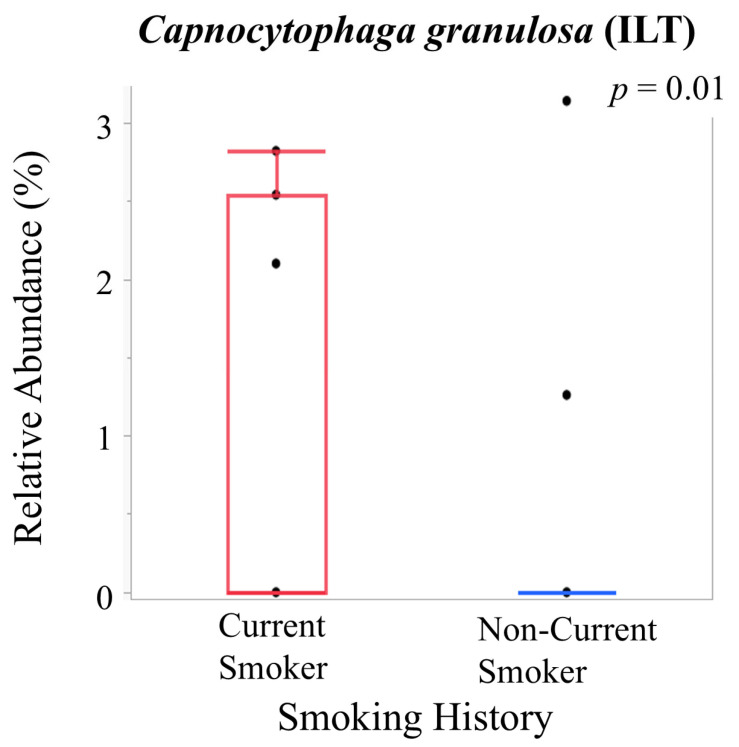
Comparison of relative abundance of bacterial species according to patient characteristics. Boxplots showing the relative abundance of bacterial species stratified by smoking history. Group comparisons were performed using the Wilcoxon rank-sum test. Statistical significance is indicated by *p*-values.

Correlations between clinical parameters and bacterial relative abundances are shown in Figure 8A,B. Group comparisons of bacterial relative abundances according to smoking history and diabetes status are presented in Figure 9 (genus level) and Figure 10 (species level).

At the genus level, *Corynebacterium* in the intraluminal thrombus showed a positive correlation with Brinkman Index (r = 0.37, *p* = 0.04), whereas *Prevotella* showed a negative correlation (r = −0.37, *p* = 0.03). *Porphyromonas* (r = 0.49, *p* = 0.01) in saliva positively correlated with the number of teeth (Figure 8A,B).

At the species level, the number of teeth positively correlated with *Fusobacterium nucleatum* (r = 0.48, *p* = 0.03) and *Leptotrichia buccalis* (r = 0.46, *p* = 0.04) in plaque (Figure 8B).

Group comparisons showed significantly higher abundance of *Capnocytophaga* in the aneurysmal wall of current smokers (*p* = 0.02) and higher *Neisseria* in the intraluminal thrombus of non-smokers (*p* = 0.01). *Porphyromonas* in the aneurysmal wall was higher in diabetic patients (*p* = 0.03) (Figure 9).

At the species level, *Capnocytophaga granulosa* was higher in the intraluminal thrombus of current smokers (*p* = 0.01) (Figure 10).

## 3. Discussion

In the present study, bacterial DNA from oral taxa, including *Prevotella*, *Leptotrichia*, *Capnocytophaga*, and *Porphyromonas,* was detected in aneurysmal wall and intraluminal thrombus samples [17,18]. The detection of bacterial DNA in the aneurysmal wall and intraluminal thrombus of patients with clinically diagnosed non-infectious AAA represents an important finding.

Although the sequencing depth of vascular samples was markedly lower than that of fecal and oral samples, bacterial DNA was reproducibly amplified, indicating the presence of trace bacterial populations in these sites.

### 3.1. Comparison with Previous Studies and Identification of Oral Bacteria

Detection of bacterial DNA in aneurysmal tissues has been reported previously [4,5]. These studies identified *Acinetobacter*, *Burkholderia*, and *Escherichia* using 16S rRNA sequencing, and *Staphylococcus*, *Bifidobacterium*, and *Clostridium* using qPCR. Furthermore, Kurihara et al. detected periodontal disease-associated bacteria such as *Porphyromonas gingivalis* and *Prevotella intermedia* from the aneurysmal wall and intraluminal thrombus by PCR. In addition, a systematic review by Salhi et al. summarized that several studies have detected DNA of oral bacteria in aneurysmal tissues using PCR. The results of the present study also support these previous findings, as bacterial DNA was detected in the aneurysmal wall and intraluminal thrombus. However, unlike those studies, our analysis identified bacterial DNA from genera including oral commensals such as *Streptococcus*, *Leptotrichia*, *Prevotella*, and *Porphyromonas* through 16S rRNA sequencing. The target region of the 16S rRNA gene analyzed in previous studies was not explicitly stated, suggesting that methodological differences, including the amplified region, may have contributed to the discrepancies in results. Moreover, the detection of DNA from specific oral commensal species, such as *Prevotella melaninogenica* and *Leptotrichia buccalis*, suggests that oral microorganisms may reach the aneurysmal wall via the bloodstream, consistent with hypotheses proposed in previous reports [13,19]. In contrast, DNA from intestinal bacteria, including *Bacteroides* and *Bifidobacterium*, was rarely detected in the aneurysmal wall or intraluminal thrombus, suggesting a possible oral origin of the detected bacterial DNA.

### 3.2. Comparison of Bacterial Diversity Between Oral and Fecal Samples

In the diversity analysis of oral and fecal samples, the α-diversity of dental plaque was significantly lower. Dental plaque is a mature biofilm firmly attached to the tooth surface, where spatial structure, nutrient gradients, and redox conditions remain relatively stable; therefore, strong environmental filtering occurs. This leads to a relatively narrow range of taxa (low α-diversity) within the plaque [20,21].

In contrast, saliva represents a composite sample of various microenvironments within the oral cavity, where local conditions fluctuate dynamically over time and through mechanical factors. Consequently, a greater variety of taxa tends to coexist in saliva [22,23].

Moreover, the intestinal tract provides a complex ecological environment that enables the growth and coexistence of diverse microorganisms, characterized by a wide range of nutrients, low oxygen conditions, and chemical gradients such as pH and bile acids. Therefore, the α-diversity of fecal samples is higher than that of oral samples, particularly dental plaque. Indeed, previous comparative studies of the human oral cavity and intestine have shown that fecal samples exhibit higher microbial diversity than oral samples (dental plaque and saliva) [24].

β-diversity analysis based on genus-level presence/absence data demonstrated that microbial community membership in aneurysmal wall and intraluminal thrombus samples showed partial similarity to oral samples rather than to fecal samples. This finding suggests that the microbial profiles detected in aneurysmal tissues may reflect the presence of bacteria originating from the oral cavity.

Oral microbiota are known to enter the systemic circulation through transient bacteremia, particularly in individuals with periodontal disease, and may subsequently colonize distant tissues. The observed similarity between aneurysmal tissues and oral samples supports the hypothesis that oral bacteria may be associated with the microbial environment within the aneurysmal lesion.

### 3.3. Detection of Periodontal Pathogen–Associated Taxa in Aneurysmal Wall and Intraluminal Thrombus

Although the present study was not designed to establish causal mechanisms, previous studies that reported the prevalence or severity of periodontitis in patients with AAA, as well as the detection of periodontal bacterial DNA in aneurysmal walls, have suggested a possible association between oral bacteria and the development of AAA or the progression of atherosclerosis [11,12,13]. Periodontal pathogens are thought to contribute to AAA formation by promoting the production of MMPs and the degradation of the ECM.

In particular, oral bacteria such as *Prevotella* and *Fusobacterium* species, which are considered to originate from the oral cavity, possess strong biofilm-forming ability and have been reported to induce inflammatory cytokines and enhance MMP expression, thereby contributing to structural disruption of the vascular wall [14,15,16]. Biofilms form a protective matrix that shields bacteria from host immune responses and antimicrobial agents, enabling persistent colonization within tissues [25]. Consequently, continuous stimulation of the innate immune system can lead to sustained production of inflammatory cytokines and tissue-degrading enzymes, such as MMPs, resulting in progressive tissue destruction and chronic inflammation [26].

In the arterial walls of AAA patients, the production of proteases—particularly MMP-2 and MMP-9—by macrophages and neutrophils has been demonstrated, leading to degradation of the ECM components, including elastin and collagen, and consequent structural failure of the arterial wall [2]. The degradation of the ECM results in loss of vascular elasticity and stability, thereby promoting the formation of the characteristic aneurysmal dilatation in AAA.

Furthermore, smoking has been reported to disrupt the oral microbial balance and impair the barrier function of the gingival epithelium [27,28]. Given that most patients in the present cohort had a history of smoking, it is plausible that smoking-induced impairment of mucosal barrier integrity and vascular endothelial dysfunction facilitated the hematogenous translocation of bacteria. In addition, *Prevotella* and *Fusobacterium* species are known anaerobes [29] that can survive and maintain metabolic activity even in the hypoxic environment characteristic of aneurysmal lesions.

In the present study, several genera including periodontal pathogen-associated taxa, such as *Leptotrichia*, *Porphyromonas*, *Prevotella*, and *Fusobacterium*, were detected in the aneurysmal wall or intraluminal thrombus. Furthermore, when these taxa were detected in aneurysm-derived samples, the same genera tended to be identified in the corresponding oral samples (dental plaque or saliva) from the same patients (Figure 5). Previous studies have demonstrated that these bacteria can translocate within the host via the bloodstream by penetrating inflamed tissues or vascular endothelium. Therefore, the detection of their DNA in the aneurysmal wall and intraluminal thrombus suggests the possibility of hematogenous dissemination from the oral cavity.

At the species level, however, differences in detection patterns among sample types were observed (Figure 7). *Prevotella melaninogenica* was detected in the corresponding oral samples in most cases in which it was identified in the aneurysmal wall or intraluminal thrombus, whereas *Prevotella pallens* was detected in oral samples in all cases in which it was present in aneurysm-derived specimens. Given that these bacteria are common oral commensals, the high concordance rates support the interpretation that *Prevotella* species detected in aneurysm-derived samples are likely of oral origin.

In contrast, *Capnocytophaga granulosa* and *Fusobacterium nucleatum* showed discordant detection in a subset of cases, in which they were identified in aneurysm-derived samples but not in the corresponding oral samples. However, these species were also not detected in fecal samples from the same patients. Considering that these bacteria are known oral commensals, their absence in oral samples may reflect technical or biological factors, including bacterial abundance below the detection limit, site-specific variability in bacterial distribution, or temporal fluctuations in the oral microbiota at the time of sampling.

### 3.4. Interpretation of Ralstonia Detection and the Possibility of Contamination

On the other hand, *Ralstonia* species, which were frequently detected in the aneurysmal wall and intraluminal thrombus, have repeatedly been reported as sources of contamination in water systems of medical devices, DNA extraction kits, and PCR reagents [30,31]. Therefore, careful interpretation is required when considering their detection as evidence of pathogenicity or local colonization. *Ralstonia pickettii* and *Ralstonia insidiosa* have often been identified from ultrapure water and sterilized reagents, and are well recognized as representative environmental or reagent contaminants in 16S rRNA and metagenomic analyses [31]. Accordingly, in the present study, additional functional investigations—such as gene expression analysis, bacterial culture, or in situ hybridization—would be necessary to discuss their physiological significance or colonization potential within the aneurysmal lesions. However, in the present study, we did not perform specific analyses to determine whether the detected *Ralstonia* originated from contamination or represented biologically relevant signals.

In addition, we acknowledge that the absence of appropriate negative controls limits our ability to fully exclude the possibility of contamination. In particular, obtaining suitable negative control samples for aneurysmal tissue is technically challenging. Therefore, the detected bacterial signals should be interpreted with caution.

### 3.5. Correlation Between Tooth Number and Oral Microbiota

We also found important associations between clinical background and bacterial profiles. Bacterial taxa such as *Fusobacterium nucleatum* and *Leptotrichia buccalis*, which positively correlated with tooth number, are anaerobic or facultatively anaerobic bacteria frequently detected in the oral cavity [32,33,34]. Increased tooth count may offer more ecological niches for these bacteria, increasing their abundance. Although *Fusobacterium nucleatum* and *Leptotrichia buccalis* are opportunistic pathogens capable of contributing to systemic infections under certain conditions, they also function as common constituents of the oral microbiota. Therefore, their presence must be interpreted in context, considering both ecological roles and potential pathogenicity.

### 3.6. Differences in Bacterial Detection Associated with Smoking

In this study, certain bacterial taxa tended to be more frequently detected depending on smoking status. Smoking is known to induce chronic inflammation of the vascular wall, leading to excessive secretion of proteases such as MMPs, which contribute to ECM degradation and VSMC apoptosis [2,35,36]. The observed negative correlation between the Brinkman Index and the relative abundance of *Prevotella* in the intraluminal thrombus suggests that smoking may suppress the colonization or proliferation of *Prevotella* species by altering the redox environment and local immune responses in the oral cavity. A Korean study similarly reported a significant reduction in the relative abundance of *Prevotella* in the oral microbiota of smokers [37], consistent with the present findings. Conversely, several studies summarized by Maki et al. have reported an increase in *Prevotella* among smokers [38].

Furthermore, in two-group comparisons, current smokers exhibited significantly higher relative abundances of *Capnocytophaga* in the aneurysmal wall and *Capnocytophaga granulosa* in the intraluminal thrombus compared with non-smokers. *Capnocytophaga* species are facultative anaerobes that predominantly inhabit subgingival plaque in patients with periodontitis [39,40], and have been implicated in infectious endocarditis, indicating their potential for hematogenous dissemination [41]. Some *Capnocytophaga* species have been reported to evade macrophage phagocytosis and inhibit their bactericidal activity [42]. Smoking-induced impairment of local immune responses, together with mucosal barrier and endothelial damage, may therefore facilitate bacterial translocation into the bloodstream through reduced phagocytic and bactericidal function of macrophages.

### 3.7. Differences in Bacterial Detection Associated with Diabetes

Furthermore, in the aneurysmal wall, the relative abundances of *Porphyromonas* and *Gemella* were significantly higher in patients with diabetes. Diabetes is known to impair innate immune function, disrupt vascular endothelial barrier integrity, and promote a state of chronic systemic inflammation [43,44], all of which may influence the composition of microbial communities, including those in the oral and intestinal environments. The high prevalence of periodontitis in diabetic patients and the sustained expression of proinflammatory cytokines at affected sites [45] may promote the local proliferation of periodontitis-associated bacteria such as *Porphyromonas*. *Porphyromonas* species, particularly *Porphyromonas gingivalis*, are strongly associated with periodontitis and are widely regarded as keystone pathogens that can impair innate immune responses and drive oral microbial dysbiosis [46,47].

### 3.8. Interplay Between Hemodynamics, Inflammation, and Microbial Factors in AAA

A growing body of evidence indicates that aneurysm pathogenesis is driven by a complex interplay between hemodynamic stress and inflammatory responses within the vascular wall. Hemodynamic forces, such as altered wall shear stress, have been shown to modulate endothelial function and promote inflammatory signaling, ultimately contributing to extracellular matrix degradation and aneurysm progression [48]. In addition, recent studies have suggested that microbial components may act as modulators of vascular inflammation, either through direct colonization or via systemic immune activation [12,13].

In this context, our findings provide descriptive evidence of bacterial DNA, including oral-associated taxa, within aneurysmal tissues. Although the present study does not directly assess hemodynamic parameters or inflammatory pathways, the detection of microbial DNA raises the possibility that bacterial components may contribute to the inflammatory milieu associated with aneurysm development.

### 3.9. Summary and Future Perspectives

Although bacterial DNA was detected in all aneurysmal samples, indicating that microbial signals were consistently present across the study cohort, we did not perform systematic analyses of associations between the presence of individual bacterial taxa and patient demographic or clinical characteristics, as such analyses would be underpowered and potentially unreliable given the limited sample size and the low variability in detection across patients. Instead, exploratory analyses focused on relative abundance and microbial composition, which provide a more quantitative assessment of microbial variation across samples.

In the present study, bacterial DNA from oral taxa, including *Prevotella*, *Leptotrichia*, *Fusobacterium*, and *Porphyromonas,* was detected in both aneurysmal wall and intraluminal thrombus samples, corresponding to bacteria present in the same patients’ oral cavities. These findings support the possibility of a translocation of oral microbiota to aneurysmal tissues. This finding provides a novel perspective on the microbial characteristics of AAA and represents a key finding of the present study.

Limitations include the low bacterial DNA yield in vascular tissues and the inability of 16S rRNA sequencing to distinguish live from dead bacteria. Thus, definitive evidence of bacterial colonization or viability was not obtained. In addition, the present study was designed as a detection-based investigation, and the findings should therefore be interpreted as descriptive observations rather than evidence of causal relationships between bacterial presence and AAA development. The small sample size also limited the statistical power to draw robust conclusions. All enrolled patients were male, preventing the evaluation of potential sex differences. Furthermore, as the study population consisted exclusively of Japanese individuals, potential racial or ethnic variations could not be assessed.

Future studies should include a larger cohort of patients and integrate clinical factors such as sex, smoking status, and the severity of periodontitis for comprehensive analysis. To clarify the viability of bacteria within the aneurysmal wall and intraluminal thrombus, combined approaches using reverse transcription quantitative PCR to assess transcriptional activity and propidium monoazide digital PCR to exclude signals from dead bacterial DNA would be valuable. Furthermore, integrating metagenomic and metatranscriptomic analyses is expected to enable comprehensive evaluation of the functional characteristics and metabolic activities of bacteria residing in aneurysmal lesions.

## 4. Materials and Methods

### 4.1. Sample Collection

Patients who underwent open surgical repair for abdominal aortic aneurysm at Tohoku University Hospital were enrolled; those treated with endovascular repair were excluded. Patients with clinical signs suggestive of infectious (mycotic) aneurysm, including fever or elevated inflammatory markers, were also excluded. The indication for surgical repair was based on standard clinical criteria in Japan, including a maximum aneurysm diameter of ≥50 mm for fusiform aneurysms. For saccular aneurysms, surgical indication was determined based on a comprehensive assessment of aneurysm morphology, size, and operative risk. Feces, saliva, and dental plaque were collected preoperatively.

This study did not include healthy vascular control samples, as obtaining aortic tissue from healthy individuals is not ethically feasible in clinical practice.

Aneurysmal wall and intraluminal thrombus samples were aseptically collected as separate specimens during open surgical repair. After opening the aneurysm sac, intraluminal thrombus was removed and branch vessels were ligated for hemostasis. Following graft replacement and confirmation of hemostasis, a portion of the aneurysmal wall was excised from a redundant area that was not required for graft coverage, regardless of the presence or distribution of thrombus, and used as the study sample. Excised tissue samples were trimmed to an appropriate size and immediately placed into sterile tubes. Intraluminal thrombus samples, removed during surgery, were independently trimmed and collected in separate sterile tubes. Fecal samples were self-collected by patients using sterile containers containing RNAlater™ (Thermo Fisher Scientific, Waltham, MA, USA). Oral samples were collected by attending dentists during preoperative dental examination. All samples were stored at −80 °C and subsequently transferred to the Tohoku University Clinical Biobank (TUCB) for further analysis. The collection of fecal, saliva, and dental plaque samples, as well as subsequent DNA extraction and microbiome analysis, were performed according to previously established protocols used by the Tohoku Medical Megabank Project [49].

This study was approved by the Institutional Review Board of Tohoku University Hospital (approval number: 2024-1-944). Written informed consent was obtained from all participants prior to sample collection.

### 4.2. DNA Extraction

DNA was extracted using the ISOSPIN Fecal DNA Kit (Nippon Gene, Tokyo, Japan) via bead-beating using FastPrep-24 (MP Biomedicals, Irvine, CA, USA), following the manufacturer’s instructions. All sample types, including aneurysmal wall and intraluminal thrombus, were subjected to the same homogenization procedure to ensure efficient disruption of both tissue and bacterial cells prior to DNA extraction. Fecal DNA was extracted at the TUCB, while DNA from other samples was extracted at the Clinical Phenome Group, Advanced Research Center for Innovations in Next-GEneration Medicine, Tohoku University. DNA quantity was assessed using absorbance (NanoDrop™ 2000c; Thermo Fisher Scientific, Waltham, MA, USA) and fluorescence-based assays (Qubit™ dsDNA HS Assay Kit or Quant-iT™ PicoGreen™ dsDNA Assay Kit; Thermo Fisher Scientific, Waltham, MA, USA).

### 4.3. 16S Library Preparation

Post-PCR procedures were performed by the Clinical Phenome Group. For feces, saliva, and dental plaque samples, 10 ng of extracted DNA was used, whereas for aneurysmal wall and intraluminal thrombus samples, 100 ng of DNA was used due to a high proportion of contaminating human DNA. The 16S rRNA gene V4 region (258–259 bp) was targeted for amplification. The V4 region was selected because it provides high phylogenetic resolution with minimal amplification bias and allows full overlap of paired-end reads on the Illumina MiSeq platform, ensuring high-quality sequence assembly. The V4 region alone was used instead of the V3–V4 combination to minimize amplification bias associated with low-biomass vascular samples, as shorter amplicons improve amplification efficiency and sequence quality under low-DNA conditions.

Library preparation was performed using a two-step PCR method as previously described [49]. For the amplicon PCR, primers with gene-specific sequences fused to Illumina overhang adapters were used as follows:

Forward primer:

5′-ACACTCTTTCCCTACACGACGCTCTTCCTCTNNNNNNGTGCCAGCMGCCGCGGTAA-3′

Reverse primer:

5′-GTGACTGGAGTTCAGACGTGTGCTCTTCCGATCTNNNNNNGGACTACHVGGGTWTCTAAT-3′

PCR was performed using Ex Taq DNA polymerase (TaKaRa Bio Inc., Kusatsu, Shiga, Japan). Thermal cycling conditions were as follows: initial denaturation at 94 °C for 3 min; 35 cycles of 94 °C for 45 s, 50 °C for 1 min, and 72 °C for 1 min 30 s; followed by a final extension at 72 °C for 10 min.

Indexing PCR was performed using primers containing 8-base dual index sequences and Illumina sequencing adapters for sample identification. Thermal cycling conditions were: initial denaturation at 98 °C for 30 s; 12 cycles of 98 °C for 40 s, 65 °C for 30 s, and 72 °C for 30 s; and a final extension at 72 °C for 5 min.

Library quality and quantification were assessed by electrophoresis using the 4200 TapeStation system (Agilent, Santa Clara, CA, USA) and by real-time PCR.

### 4.4. Sequencing

Sequencing was performed using the MiSeq system (Illumina, Inc., San Diego, CA, USA). A 300 bp paired-end sequencing protocol was conducted using the MiSeq Reagent Kit v3 (Illumina, Inc., San Diego, CA, USA), with 50% PhiX added according to the manufacturer’s instructions.

### 4.5. Sequence Data Analysis

Raw paired-end reads were demultiplexed and processed using QIIME2 (version 2023.4; University of Arizona, Tucson, AZ, USA). Reads were denoised, merged, and chimera-filtered using the DADA2 plugin to generate ASVs. Detailed QIIME2 commands used for sequence processing, including DADA2 denoising and taxonomic assignment, are provided in the Supplementary Materials. Detection of bacterial taxa was based on high-quality reads obtained after DADA2 filtering. No explicit read-count threshold was applied for determining the presence or absence of bacterial taxa; instead, detection was defined by the presence of assigned ASVs in each sample. Representative sequences were taxonomically assigned based on the V4 region using a QIIME2-implemented classifier trained on a curated 16S reference database. Subsequently, diversity analyses were performed for each sample type. For each bacterial taxon, both presence/absence and relative abundance were evaluated using all sequencing reads obtained to characterize overall community structure and taxon-specific distribution patterns across samples.

The relative abundance of each bacterial taxon was calculated by dividing the number of reads assigned to each taxon by the total number of reads per sample after quality filtering.

α-diversity analyses were performed on samples containing at least 10,000 effective reads. This threshold, commonly adopted in previous studies, was selected to ensure the reliability of diversity assessments and the sensitivity of taxonomic detection [49,50,51]. Faith’s PD was calculated to estimate α-diversity. In contrast, β-diversity analysis was performed using all available samples, including low-biomass vascular specimens, to enable comparison across all sample types. β-diversity was evaluated by PCoA based on Jaccard distance, which is less affected by sequencing depth. β-diversity analysis was performed at the genus level. β-diversity analyses based on Jaccard distance were performed using Python (version 3.12.13, Google Colab environment). Distance matrices were calculated using the scikit-bio package (version 0.7.2), and principal coordinate analysis (PCoA) was conducted based on these distance matrices. Group differences were assessed using PERMANOVA with 9,999 permutations. Detailed Python scripts used for β-diversity analysis, including Jaccard distance calculation, PCoA, and PERMANOVA, are provided in the Supplementary Materials.

### 4.6. Statistical Analysis

Proportions and medians with IQRs were calculated to describe patient backgrounds. Associations between clinical variables and the relative abundance of bacterial taxa were evaluated. For non-normally distributed data, the Wilcoxon rank-sum test was used for two-group comparisons, and Spearman’s rank correlation coefficient (r) was used for correlation analysis between continuous variables. Because of the limited sample size, adjustment for potential confounding factors was not performed in the two-group comparisons. The Kruskal–Wallis test was used to compare Faith’s PD among groups. When overall differences were significant, post hoc multiple comparisons were performed using the Steel–Dwass method to identify pairwise differences. A *p*-value < 0.05 was considered statistically significant. All statistical analyses were performed using JMP Pro 17.2.0 (SAS Institute Inc., Cary, NC, USA).

## 5. Conclusions

In conclusion, this study identified bacterial DNA, including oral-associated taxa, in the aneurysmal wall and intraluminal thrombus of patients with AAA. Notably, oral-associated genera such as *Prevotella* were detected in a substantial proportion of aneurysmal samples (approximately 80%). These findings demonstrate the presence of microbial DNA in aneurysmal tissues and provide descriptive evidence of microbial signatures in AAA. Further studies are required to clarify the biological relevance and potential clinical implications of these microbial signals.

## Figures and Tables

**Table 1:** Demographics and Clinical Characteristics of the Study Population.

Overall	N = 32
Variable	No. (%) or median (IQR)
Demographics	
Age, y	70 (65–75)
Male	32 (100)
Diameter of aneurysm	50 (50–57)
Fusiform aneurysm	28 (88)
Current and Ex-smoker	31 (97)
Current smoker	7 (22)
Brinkman Index	755 (320–1042)
eGFR, mL/min/1.73 m^2^	57 (46–65)
Number of teeth	20 (0–27)
Comorbidities	
Hypertension	25 (78)
Diabetes	7 (22)
Dyslipidemia	22 (69)
Periodontitis	24 (75)
Oral medication	
Acid-suppressing agents	15 (47)

All values at the time of treatment.

**Table 2:** Number of Samples by Specimen Type.

Sample	Sample No.
Aneurysmal wall	32
Intraluminal Thrombus	31
Feces	28
Plaque	20
Saliva	24

## Data Availability

The data are not publicly available due to institutional and ethical restrictions but may be available from the corresponding author upon reasonable request.

## Supplementary Materials

The following supporting information can be downloaded at: https://www.mdpi.com/article/10.3390/ijms27104396/s1. Detailed QIIME2 commands used for sequence processing, including DADA2 denoising and taxonomic assignment, as well as Python scripts for β-diversity analysis (Jaccard distance calculation, PCoA, and PERMANOVA), are provided.

## Abbreviations

The following abbreviations are used in this manuscript:

AAA	Abdominal Aortic Aneurysm
ASV	Amplicon Sequence Variant
AW	Aneurysmal wall
ECM	Extracellular Matrix
Faith’s PD	Faith’s Phylogenetic Diversity
ILT	Intraluminal thrombus
IQR	Interquartile Range
MMP	Matrix Metalloproteinase
PCoA	Principal coordinate Analysis
qPCR	Real-time quantitative Polymerase Chain Reaction
PERMANOVA	Permutational multivariate analysis of variance
rRNA	ribosomal RNA
TUCB	Tohoku University Clinical Biobank
VSMC	Vascular Smooth Muscle Cell
