# Supplementary material for: Detection of Oral Bacterial DNA in Abdominal Aortic Aneurysm and Its Microbial Associations
Source: Int J Mol Sci. 2026 May 14;27(10):4396. doi: 10.3390/ijms27104396 (PMC13207979; doi:10.3390/ijms27104396)
Supplement: Supplementary file 1 [file ijms-27-04396-s001.zip › ijms-4227013-supplementary.pdf]

S1. QIIME2 Commands for Sequence Processing

```
FORWARD_PRIMER=GTGCCAGCMGCCGCGGTAA
REVERSE_PRIMER=GGACTACHVGGGTWTCTAAT
DIVERSITY_READ_NUMBER=10000

conda activate qiime2-2023.2

qiime tools import ¥
   --type 'SampleData[PairedEndSequencesWithQuality]' ¥
   --input-path manifest.tsv ¥
   --output-path paired-end-demux.qza ¥
   --input-format PairedEndFastqManifestPhred33V2

qiime cutadapt trim-paired ¥
   --i-demultiplexed-sequences paired-end-demux.qza ¥
   --o-trimmed-sequences trimmed-paired-end-demux.qza ¥
   --p-front-f ${FORWARD_PRIMER} ¥
   --p-front-r ${REVERSE_PRIMER} ¥
   --p-discard-untrimmed true ¥
   --p-cores 8

qiime dada2 denoise-paired ¥
   --i-demultiplexed-seqs trimmed-paired-end-demux.qza ¥
   --o-table table.qza ¥
   --o-representative-sequences rep-seqs.qza ¥
   --o-denoising-stats denoising-stats.qza ¥
   --p-trim-left-f 0 ¥
   --p-trunc-len-f 220 ¥
   --p-trim-left-r 0 ¥
   --p-trunc-len-r 200 ¥
   --p-n-reads-learn 100000 ¥
   --p-n-threads 0 ¥
   --verbose

qiime phylogeny align-to-tree-mafft-fasttree ¥
   --i-sequences rep-seqs.qza ¥
```

```
    --output-dir phylogeny-align-to-tree-mafft-fasttree ¥
    --p-n-threads auto ¥
    --verbose

qiime diversity core-metrics-phylogenetic ¥
    --i-phylogeny phylogeny-align-to-tree-mafft-fasttree/rooted_tree.qza ¥
    --i-table table.qza ¥
    --m-metadata-file metadata.tsv ¥
    --p-sampling-depth ${DIVERSITY_READ_NUMBER} ¥
    --output-dir core-metrics-results_${DIVERSITY_READ_NUMBER}

qiime feature-classifier classify-sklearn ¥
    --i-classifier gg_2022_10_backbone.v4.nb.qza ¥
    --i-reads rep-seqs.qza ¥
    --o-classification taxonomy-greengenes2.qza ¥
    --p-confidence 0.7 ¥
    --p-n-jobs 8
```

S2. Python Script for β-Diversity Analysis

```
# Python-based beta diversity analysis

import pandas as pd
import matplotlib.pyplot as plt
from skbio.diversity import beta_diversity
from skbio.stats.ordination import pcoa
from skbio.stats.distance import permanova

# Load genus-level presence/absence table
# The input file contained one row per sample.
# "SampleType" indicated specimen type, and the remaining columns indicated bacterial genera.
df = pd.read_csv("Genus_presence_absence.csv")

# Assign sample IDs
df.index = [f"S{i+1}" for i in range(len(df))]
```

```
# Extract sample groups
groups = df["SampleType"].astype(str)

# Prepare genus-level presence/absence matrix
X = df.drop(columns=["SampleType"])
X = X.apply(pd.to_numeric, errors="coerce").fillna(0)
X = (X > 0).astype(int)

# Calculate Jaccard distance matrix
dm = beta_diversity(
    metric="jaccard",
    counts=X.values,
    ids=X.index.astype(str)
)

# Principal coordinate analysis
ord_res = pcoa(dm)

# PERMANOVA
ids = list(dm.ids)
permanova_results = permanova(
    distance_matrix=dm,
    grouping=groups.loc[ids],
    permutations=9999
)

print(permanova_results)

# Generate PCoA plot
coords = ord_res.samples.loc[list(dm.ids), ["PC1", "PC2"]].copy()
coords["SampleType"] = groups.loc[list(dm.ids)].values

plt.figure(figsize=(6, 5))

for group_name, subset in coords.groupby("SampleType"):
    plt.scatter(
```

```
            subset["PC1"],
            subset["PC2"],
            label=group_name,
            alpha=0.8
        )

plt.xlabel(f"PC1 ({ord_res.proportion_explained['PC1']:.1%})")
plt.ylabel(f"PC2 ({ord_res.proportion_explained['PC2']:.1%})")
plt.legend(bbox_to_anchor=(1.02, 1), loc="upper left")
plt.tight_layout()
plt.savefig("PCoA_Jaccard_genus_presence_absence.png", dpi=300)
plt.close()
```
